# Gastric polyps: a 10-year analysis of 18,496 upper endoscopies

**DOI:** 10.1186/s12876-022-02154-8

**Published:** 2022-02-19

**Authors:** Haythem Yacoub, Norsaf Bibani, Mériam Sabbah, Nawel Bellil, Asma Ouakaa, Dorra Trad, Dalila Gargouri

**Affiliations:** 1 Gastroenterology and Hepatology Department, Habib Thameur Hospital, Tunis, Tunisia; 2 Faculty of Medicine of Tunis, El Manar University, Tunis, Tunisia

**Keywords:** Stomach, Polyp, Polypectomy, Endoscopic mucosal resection

## Abstract

**Background/aims:**

Gastric polyps (GPs) are usually asymptomatic lesions of the upper gastrointestinal tract observed in 1–3% of esophagogastroduodenoscopies (EGD). Most GPs are benign. The aim of this study was to precise the frequency of different types of gastric polyps in our population, and to analyze their possible association with other factors.

**Materials and methods:**

A total of 18,496 consecutive patients undergoing EGD over a 10-year period (between 2007 and 2018) in a tertiary hospital were retrospectively reviewed. Eighty-six patients diagnosed with gastric polyps were analysed. Demographics, medical history of the patients, and indication for gastroscopy were collected. Morphological, histological characteristics of polyps, and therapeutic management data were also collected.

**Results:**

GPs were found in 86 out of 18,496 (0.46%) reviewed EGD, corresponding to a total of 141 polyps. There were 64 female (74.4%) and 22 male patients (25.6%) with a sex ratio (M/F) of 0.34. The average age was 58.1 years. One hundred and forty one polyps were included, and histopathology was obtained on 127 GPs. The most common location was the fundus (59.6%) and 48.9% were smaller than 5 mm. The polyp was unique in 75.6% of cases. According to Paris classification, 80% of the polyps were sessile (Is). Hyperplastic polyps were the most common (55.9%), followed by sporadic fundic gland polyps observed in 23 patients (18.1%), 7 (5.5%) were adenomas and 4 (3.1%) were neuroendocrine tumors type 1. The following factors were associated with hyperplastic polyps: anemia (*p* = 0.022), single polyp (*p* = 0.025) and size ≥ 5 mm (*p* = 0.048). Comparing hyperplastic polyps’ biopsies to resected polyps, no difference was found in the evolutionary profile of the 2 groups. A size less than 10 mm (*p* = 0.013) was associated with fundic gland polyps. Sixty polyps (47.2%) were treated by cold forceps, 19 (15%) treated by a mucosal resection and 15 (11.8%) with diathermic snare. Five procedural bleeding incidents were observed (3.9%). Only the use of anticoagulant treatment was associated with a high bleeding risk (*p* = 0.005). The comparative histological study between specimens of biopsied GPs and endoscopic polypectomy led to an overall agreement of 95.3%.

**Conclusion:**

In our study, the GPs frequency was 0.36%. Hyperplastic polyps and fundic gland are the most common in our country. The high frequency of *Helicobacter pylori* infection in our patients and in our area may explain the high frequency of HP.

## Introduction

Gastric polyps (GPs) are defined as luminal projections above the plane of the adjacent mucosa regardless of its histological type [1]. Gastric polyps are usually discovered incidentally during esophagogastroduodenoscopies (EGD) and their prevalence is estimated from 0.5 to 23% of all upper gastrointestinal endoscopies [2]. Some polyps can occasionally present with bleeding, anemia, or gastric outlet obstruction [3].

The majority of polyps are benign (> 85% of cases). The risk of malignancy or malignant transformation of gastric polyps depends on their histological nature. GPs have been associated with multiple factors, such as *H. pylori* infection for hyperplastic polyps and adenomas, proton-pump inhibitor (PPI) use for fundic gland polyps [4, 5].

The aim of this study was to precise the frequency of different types of gastric polyps in our population and to analyze their possible association with other factors to evaluate the results of curative endoscopic resection of gastric polyps and to study the evolutionary status of unresected gastric polyps.

## Methods

### Study design

A retrospective study in which all consecutive patients with GPs were enrolled was performed at a tertiary-level hospital (Habib Thameur Hospital of Tunis) from 2008 to 2017. A total of 18,496 consecutive EGD over a 10-year period were retrospectively reviewed. Eighty-six patients diagnosed with gastric polyps were analysed. Follow-up gastroscopies performed on the same patient were not excluded. This study was performed according to the Declaration of Helsinki, following the guidelines for good clinical practice. Habib Thameur Hospital ethics committee approved the study protocol.

All cases of gastric polyps were identified from endoscopy reports. All data regarding patients were obtained from the electronic medical record. Demographic data (sex, age), relevant pathological history (colorectal cancer or hereditary polyposis syndrome, colon polyp, cirrhosis), routine hemograms, as well as data related to the EGD indication of gastroscopy, number and size of GPs, location, histological type, and the presence of chronic gastritis or *H. pylori* infection using the Hematoxylin eosin staining) were collected. The polyp size was estimated by comparing it with the opening size of the used biopsy forceps. In patients with multiple polyps, we collected the endoscopic characteristics of the four largest polyps. GP recurrence following resection were also collected (number, location, size, histological type and, recurrence interval after polypectomy) Patients whose hemoglobin levels were less than 13 g/dl in males and 12 g/dl in females were considered with anemia.

### Ethics approval and consent to participate

This study was performed according to the Declaration of Helsinki, following the guidelines for good clinical practice. “Habib Thameur Hospital ethics committee” approved the study protocol. All methods were carried out in accordance with relevant guidelines and regulations. Informed consent to participate in the study was obtained from participants.

### Statistical analysis

The data were analyzed on the Statistical Package for the Social Sciences (SPSS) version 23, IBM SPSS Inc.; Chicago, IL, USA). Results for the continuous variables that followed a normal distribution were expressed as mean ± standard deviation and range while variables that did not follow a normal distribution were presented in median and the interquartile range.

For comparisons, Student’s *t*-test was used for quantitative variables. A univariate analysis was conducted to identify the possible associated factors with the different histological types of GPs. A multivariate analysis was carried out with variables that achieved statistical significance. The level of statistical significance was established with a *p value* ≤ 0.05.

## Results

**Fig. 1:**
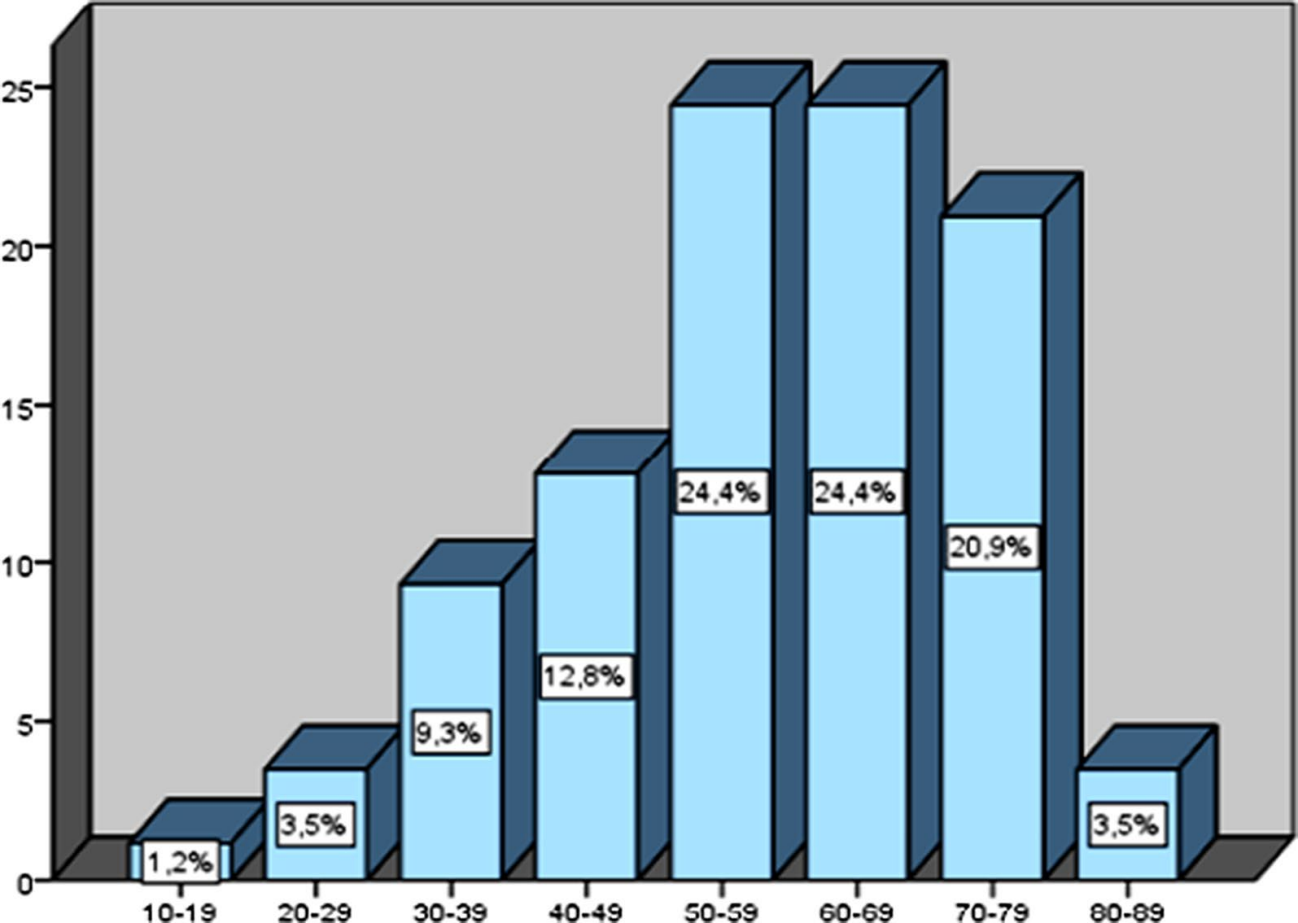
The age distribution of patients with gastric polyps

A total of 18.496 patients who underwent EGD in our endoscopy unit were included in our study. GPs were found in 86 patients corresponding to 141 GPs. We found the incidence of GP as 0.46%. The Table 1 shows the characteristics of patients with GPs. The mean age was 58.1 years and the majority were female (74.4%). Epigastric pain was the most common symptom, observed in 30 (34.9%) of the cases followed by anemia, dyspepsia and PHT monitoring in respectively 21 (24.4%), 16 (18.6%) and 13 patients (15.1%). Of the patients, 14% were under the age of 40 years, 61.6% were 40–69 years, and 24.4% were > 70 years. Age distribution of the patients with GPs was summarized in Fig. 1.

**Table 1:** Characteristics of the 86 patients with GP

Parameter	n (%)
Age (median years),(range, years)	58.1 ± 15.4, (18–84)
***Gender***	
Male	22 (25.6)
Female	64 (74.4)
***Personal history***	
GERD	26 (30.2)
Anemia	36 (44.2)
Colon polyps	2 (2.3)
Cirrhosis	11 (12.8)
Gastrectomy	3 (3.5)
Hereditary polyposis syndrome	0 (0)
***Indication***	
Epigastric pain	30 (34.9)
Dyspepsia	16 (18.6)
Anemia	21 (24.4)
UGIB	2 (2.3)
Monitoring of PHT	13 (15.1)
Other	4 (4.7)

*GP* gastric polyps, *GERD* gastro-esophageal reflux disease, *PHT* portal hypertension, *UGIB* upper gastrointestinal bleeding

More than the three-quarters of the patients had single polyps. The average polyp diameter was 6 mm (range: 2–30 mm). The diameters of the polyps were < 5 mm in 69 of cases (48.9%), 5–9 mm in 53 (37.6%) patients, 10–19 mm in 15 (10.7%) patients, and ≥ 20 mm in 4 (2.8%) patients (Table 2). The location of GPs was based on endoscopic findings; the most common localization for gastric polyps was the fundus, followed by the antrum and the corpus (Table 2).

**Table 2:** Morphological and histological characteristics of the 141 polyps

Parameter	n (%)
**Patient with GPs**	**86 (100)**
Single	65 (75.6)
Multiple	21 (24.4)
***Location***	
Fundus	51 (59.3)
Body	5 (5.8)
Antrum	28 (32.6)
Multiple location	2 (2.3)
***Size in mm***	
1–4 mm	69 (48.9)
5–9 mm	53 (33.6)
10–14 mm	8 (5.7)
15–19 mm	7 (5)
> 20 mm	4 (2.8)
***Paris classification***	
Ip	17 (12)
Is	112 (80)
IIa	11 (8)
IIb, IIc	0 (0)

*GP* gastric polyps

Histopathologic diagnosis of polyps was obtained for 127 polyps. The histological study showed hyperplastic polyps in 71 of the polyps (55.9%), followed by fundic gland polyps (n = 23, 18.1%) (Table 3). The “other” category included pancreatic heterotopias, lipoma and polypoid foveolar hyperplasia.

**Table 3:** Histological analysis of the 127 GP

Histological type	n (%)
Hyperplastic	71 (55.9)
Fundic gland polyps	23 (18.1)
Adenoma	7 (5.5)
Neuroendocrine neoplasia	4 (3.1)
Xanthelasma	1 (0.8)
Inflammatory fibroid polyp	9 (7.2)
No true polyp	7 (5.5)
Other	5 (3.9)

*GP* gastric polyps

*H. pylori* infection identification was carried out with the hematoxylin eosin staining in 64 patients (patients with gastric mucosa abnormalities). *H. pylori* infection was detected in 45 patients (62.3%). *H. pylori* was positive in 30 of the 49 (61.2%) of patients with hperplastic polyps.

The factors independently associated with hyperplastic polyps were the presence of anemia, being a single polyp, and sized ≥ 5 mm. The associated variable for fundic gland polyps, was only size < 5 mm (Tables 4, 5, 6). Our data do not support a relationship between PPI and fundic gland polyps.

**Table 4:** Univariate analysis of associated factors with hyperplastic polyps (n = 127)

Parameter	Hyperplastic polyps	Non-hyperplastic polyps	*p* value
	n (%)	n (%)	
Age (years)	57.7 ± 13.4	58.8 ± 17.4	0.7
*Gender*			0.7
Male	12 (24.5)	8 (27.6)	
Female	37 (75.5)	21 (72.4)	
*Single/multiple*			**0.05**
Single	40 (81.6)	11 (38)	
Multiple	9 (18.4)	18 (62)	
*GERD*			0.7
Yes	15 (30.6)	10 (34.5)	
No	34 (69.4)	19 (65.5)	
*Anemia*			**< 10**^**–3**^
Yes	28 (57.2)	3 (10.3)	
No	21 (42.8)	26 (89.7)	
*Location*			**0.009**
Antrum	34 (47.9)	43 (76.8)	
Non antrum	37 (52.1)	13 (23.2)	
*Size in mm*			**0.002**
< 5 mm	30 (42.3)	39 (69.6)	
≥ 5 mm	41 (47.7)	17 (30.4)	
*Paris classification*			
Ip	10 (14)	5 (8.9)	0.371
Is	58 (81.7)	43 (76.8)	0.496
IIa	3 (4.3)	8 (14.3)	0.055
IIb, IIc	0 (0)	0 (0)	–

*GP* gastric polyps, *GERD* gastro-esophageal reflux disease

Significant *p* value < 0.05 are in bold

**Table 5:** Univariate analysis of associated factors with fundic gland polyps (n = 127)

Parameter	Fundic gland polyps	Non-fundic gland polyps	*p* value
	n (%)	n (%)	
Age (years)	53.3 ± 21.3	58.8 ± 14.1	0.303
*Gender*			0.289
Male	1 (11.1)	19 (27.5)	
Female	8 (88.9)	50 (72.5)	
*Single/multiple*			0.170
Single	5 (55.6)	53 (76.8)	
Multiple	4 (44.4)	16 (23.2)	
*GERD*			0.7
Yes	5 (55.6)	20 (29)	
No	4 (44.4)	49 (71)	
*Anemia*			0.115
Yes	8 (88.9)	43 (62.3)	
No	1 (11.1)	26 (37.7)	
*Location*			
Fundus	23 (100)	56 (53.9)	
Non fundus	0 (0)	48 (46.1)	
*Size in mm*			**0.013**
< 5 mm	14 (60.9)	55 (53)	
≥ 5 mm	9 (39.1)	49 (47)	
*Paris classification*			
Ip	3 (13)	12 (11.5)	0.524
Is	16 (69.5)	85 (81.7)	0.273
IIa	4 (17.5)	7 (6.8)	0.105
IIb, IIc	0 (0)	0 (0)	–

GP: gastric polyps, GERD: gastro-esophageal reflux disease

Significant *p* value < 0.05 are in bold

**Table 6:** Risk value for the significant variables in the multivariate analysis

Variable	Odds ratio 95% CI	*p* value
*Hyperplastic polyps*		
Anemia	4.28 (1.39–13.17)	0.022
Single	2.85 (0.95–8.59)	0.025
Size ≥ 5 mm	1.85 (1.29–2.67)	0.048
*Fundic gland polyps*		
Size < 5 mm	2.31 (1.37–4.11)	< 0.001

*GP* gastric polyps, *GERD* gastro-esophageal reflux disease

**Fig. 2:**
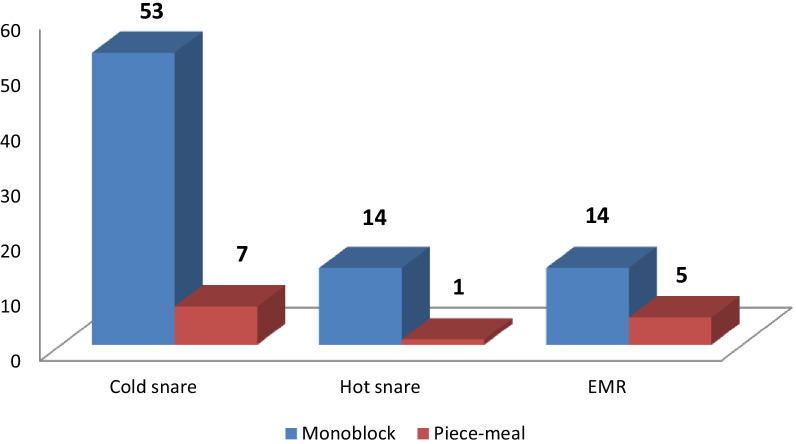
The distribution of polypectomy. *EMR* endoscopic mucosal resection

Monoblock resection was associated with polyps size less than 15 mm (*p* = 0.034) and localization in the antrum (0.013). Polypectomy with cold snare was performed in 60 patients. Polypectomy with hot snare was performed alone or in combination with endoloop treatments was performed in 15 patients. The distribution of different methods of polypectomy is given in Fig. 2.

The univariate analysis of the occurrence of bleeding incidents according to the gender, age and clinical characteristics of the patients did not show a significant difference between the two groups of patients (those with or without bleeding complication).

Anticoagulant or anti-aggregating medication was significantly correlated with the onset of bleeding (*p* = 0.002).

Twenty-one polyps were biopsied and then resected. A comparison of the histological results between biopsy specimen and polypectomy was made.

Adenomas analysis showed a 100% agreement between primary and final results. The agreement rate was 93% for hyperplastic polyps (13 polyps out of 14). The comparative histological study between specimens of biopsied GPs and endoscopic polypectomy led to an overall agreement of 95.3% (Table 7).

**Table 7:** Agreement between polypectomy and biopsy specimen in different histological types

**Histological type**	**Biopsy specimen (n)**	**Polypectomy (n)**
***Adenomas***		
With low grade dysplasia	4	4
With high grade dysplasia	1	1
**Fundic gland polyps**	1	1
**Hyperplastic polyps**	13	14
**Type I neuroendoscrine tumor**	1	1
**Inflammatory mucosa**	1	0

## Discussion

One hundred and twenty-seven specimens, corresponding to eighty-six patients, of the total of 18,496 upper gastrointestinal endoscopic procedures, taken from gastric polypoid lesions (0.46%) were reported. In the literature, a great variability was observed in the prevalence of GPs, ranging from 0.5 to 6.35% [2, 6, 7]. In our study, the prevalence of GPs is lesser than reported in literature. This can be explained by the fact that follow-up EGD performed on the same patient was not excluded.

This is the first study that evaluates the GPs frequency in Tunisia. In our study, we found that the most common symptoms in patients with GPs were epgastric pain and anemia. We also found that GPs were localized mostly in the fundus, and mostly Is according to Paris classification and the hyperplastic type was the most common.

Hyperplastic polyps and fundic gland polyps together make up to 90% [6–8] followed by adenomas and other histological type, which are much less common. These rates are similar to those observed in our population with a predominance of hypeplastic type.

Argüello et al. reported the frequency of GP as 42.8% for hyperplastic polyps, and 37.7% for fundic gland polyps. The mean age of the patients was 65.6 years and 38% were males [9]. Carmack et al. found the incidence of GP as 6.3% in 121.564 EGD. Fundic gland polyps were the most frequent polyp type, which accounted for 77% of all polyps of all polyps [6]. Fundic gland polyps were the second most common type (18.1%) of GPs lesions in our study. In the majority of series, hyperplastic polyps are the most common [9–13]. It has been suggested that the prevalence of hyperplastic polyps could be related to the high prevalence of *H. pylori* infection in our population (62.3%). Freeman et al. reported tendency of fundic gland polyps to arise in *H. pylori* -free stomachs [14] (OR 0.007, 95% CI 0.003–0.015). The same findings were also reported in Carmack et al. study (OR 0.007, 95% CI 0.003–0.016) [6]. Fundic gland polyps tend also to arise in patients who receive long-course PPI treatment [14, 15]. The widespread of PPIs use and the low *H. Pylori* infection rate may be the most important reasons behind the large frequency of fundic gland polyps reported in American studies [6, 14]. Although in three Spanish series, hyperplastic polyps were the most common which is comparable to our study [8, 9, 16].

Hyperplastic polyps are associated with chronic gastritis such as *H. pylori* gastritis, and particularly autoimmune gastritis. Patients with hyperplastic polyp have an increased risk of gastric adenocarcinoma [1, 17, 18].

In our study, adenomas were detected in seven patients (5.5%). The majority of cases we reported were low-grade intestinal-type. These polyps constitute less than 10% and have a malignant potential. They are more common in communities where gastric cancer is frequent [19]. Malignant potential of adenomas is variable (6.8% − 55.3%) [20]. Risk factors for malignancy transformation are: high-grade dysplasia, and size of the lesion [19].

It has been reported that between 16 and 37.5% of cases, and despite the endoscopic appearance of a polyp, the final histological study shows normal mucosa [6, 16]. In our study the percentage of biopsies with normal mucosa was 5.5%.

Although the majority of GP do not cause symptoms, they can be the cause of bleeding and gastric obstruction. Frequently, GP are detected during EGD performed to study gastrointestinal symptoms not attributable to polyps or asymptomatic patients examined for other reasons [6, 21].

In our study, an association between hyperplastic polyps and anemia, single polyps and size > 5 mm. It has been described in the literature between anaemia and hyperplastic polyps, while the gastrointestinal reflux was associated with fundic gland polyps [22].

A total of 94 polyps were resected with snare. Five patients had hemorrhage requiring endoscopic treatment and bleeding was controlled by endoscopic procedures. Perforation did not occur in any of our patients. In the literature, bleeding as a complication of gastric polypectomy was reported in 3.5% [23].

Relationship between long-term PPIs use and fundic gland polyps’ occurring has not yet been fully established. Jalving et al. [4] found in their study a significant association only in the subgroup of patients treated with PPI for over 1 year. Our data do not support a relationship between PPI and fundic gland polyps.

In patients with GP, evaluating *H. pylori* infection state by obtaining biopsies of the surrounding gastric mucosa is recommended and treatment is required if present [24, 25].

Hyperplastic polyps should be biopsied according to the British society of gastroenterology and an examination of the whole stomach should be made. *H pylori* infection should be detected and eradicated when present [24]. GP of the non-adenomatous type are at a low risk of malignant transformation, therefore endoscopic resection is not necessary.

Guidelines on management of hyperplastic polyps, recommend resection of polyps greater than 5 mm [26, 27].

Complete removal of the adenoma should be performed when safe to do according to the British recommendations [24].

Polypectomy is not required for sporadic fundic gland polyps. Biopsy of probable fundic gland polyps is recommended to exclude dysplasia. In patients with multiple fundic gland polyps who are under 40 years-old, or where biopsies specimens show dysplasia, colonoscopy should be performed to exclude familial adenomatous polyposis [24].

Szaloki et al. reported that there were important disagreements in 12 cases of examined forceps biopsy specimens. In 14 neoplastic, and 1 hyperplastic polyps, the degree of dysplasia seen on histological examination of the forceps biopsy specimens differed from that observed for the resected specimens. Complete agreement between the histological results on ectomized polyp, and the forceps biopsy was observed in only 55.3% of the cases [28]. In our study, Adenomas analysis showed a 100% agreement between primary and final results. The agreement rate was 93% for hyperplastic polyps (13 polyps out of 14). The overall agreement was of 95.3%.

Our study included the greatest number of EGD with patients diagnosed with GP in our country.

## Conclusion

Gastric polyps’ frequency in our study was low (0.46%). Hyperplastic polyps are the most common gastric polyps in our country. In case of single polyps, biopsies are recommended to rule out a diagnosis of adenoma or hyperplastic polyps with dysplasia. Good knowledge of practical guidelines is important for the management of GP.

## Data Availability

The data that support the findings of this study are available on request from the corresponding author, [HY]. The data are not publicly available due to [restrictions e.g. their containing information that could compromise the privacy of research participants].
